# Clarifying the reliability paradox: poor measurement reliability attenuates group differences

**DOI:** 10.3389/fpsyg.2025.1592658

**Published:** 2025-10-15

**Authors:** Povilas Karvelis, Andreea O. Diaconescu

**Affiliations:** ^1^Krembil Centre for Neuroinformatics, Centre for Addiction and Mental Health (CAMH), Toronto, ON, Canada; ^2^Department of Psychiatry, University of Toronto, Toronto, ON, Canada; ^3^Institute of Medical Sciences, University of Toronto, Toronto, ON, Canada; ^4^Department of Psychology, University of Toronto, Toronto, ON, Canada

**Keywords:** reliability paradox, test-retest reliability, individual differences, group differences, group effects, measurement reliability, effect size attenuation, clinical translation

## Abstract

Cognitive sciences are grappling with the reliability paradox: measures that robustly produce within-group effects tend to have low test-retest reliability, rendering them unsuitable for studying individual differences. Despite the growing awareness of this paradox, its full extent remains underappreciated. Specifically, most research focuses exclusively on how reliability affects correlational analyses of individual differences, while largely ignoring its effects on studying group differences. Moreover, some studies explicitly and erroneously suggest that poor reliability does not pose problems for studying group differences, possibly due to conflating within- and between-group effects. In this brief report, we aim to clarify this misunderstanding. Using both data simulations and mathematical derivations, we show how observed group differences get attenuated by measurement reliability. We consider multiple scenarios, including when groups are created based on thresholding a continuous measure (e.g., patients vs. controls or median split), when groups are defined exogenously (e.g., treatment vs. control groups, or male vs. female), and how the observed effect sizes are further affected by differences in measurement reliability and between-subject variance between the groups. We provide a set of equations for calculating attenuation effects across these scenarios. This has important implications for biomarker research and clinical translation, as well as any other area of research that relies on group comparisons to inform policy and real-world applications.

## 1 Introduction

An influential paper by Hedge et al. (2018) has highlighted the “reliability paradox”: cognitive tasks that produce robust within-group effects tend to have poor test-retest reliability, undermining their use for studying individual differences. Many studies have followed, demonstrating the prevalence of low test-retest reliability and emphasizing its implications for studying individual differences across various research contexts, including neuroimaging, computational modeling, psychiatric disorders, and clinical translation (Enkavi et al., 2019; Elliott et al., 2020, 2021; Fröhner et al., 2019; Nikolaidis et al., 2022; Kennedy et al., 2022; Nitsch et al., 2022; Blair et al., 2022; Zuo et al., 2019; Milham et al., 2021; Feng et al., 2022; Haines et al., 2023; Parsons et al., 2019; Hedge et al., 2020; Enkavi and Poldrack, 2021; Zorowitz and Niv, 2023; Gell et al., 2023; Rouder et al., 2023; Karvelis et al., 2023, 2024; Clayson, 2024; Vrizzi et al., 2025).

However, the studies on this topic tend to focus exclusively on how test-retest reliability affects correlational individual differences analyses without making it clear that it is just as relevant for studying group differences (although see LeBel and Paunonen, 2011; Zuo et al., 2019). Not only that, some studies incorrectly suggest that poor test-retest reliability is not problematic for studying group differences. For example: “*Low reliability scores are problematic only if we were interested in differences between individuals (within a group) rather than between groups*” (De Schryver et al., 2016); “*although improved reliability is critical for understanding individual differences in correlational research, it is not very relevant or informative for studies comparing conditions or groups*” (Zhang and Kappenman, 2024); “*On a more positive note, insufficient or unproven test-retest reliability does not directly imply that one cannot reliably assess group differences (e.g., clinical vs. control)*” (Schaaf et al., 2023); “*while many cognitive tasks (including those presented here) have been well validated in case-control studies (e.g., comparing MDD and healthy individuals) where there may be large group differences, arguably these tests may be less sensitive at detecting individual differences*” (Foley et al., 2024); “*The reliability paradox... implies that many behavioral paradigms that are otherwise robust at the group-level (e.g., those that produce highly replicable condition- or group-wise differences) are unsuited for testing and building theories of individual differences*” (Haines et al., 2020); “*Many tasks clearly display robust between-group or between-condition differences, but they also tend to have sub-optimal reliability for individual differences research*” (Parsons et al., 2019). Sometimes the opposite mistake is made by suggesting that poor reliability is equally detrimental for studying both between-group differences and within-group effects (e.g., see Figure 1 in Zuo et al., 2019).

An apparent common thread across these examples is the conflation of within-group effects and between-group effects, treating both simply as “group effects.” However, within- and between-group effects are often in tension. If an instrument is designed to produce strong within-group effects (i.e., robust changes across conditions or time points), it will typically do so by minimizing between-subject variability – which in turn reduces its ability to reliably detect individual or between-group differences. This trade-off lies at the heart of the reliability paradox. The key insight here is that both group and individual differences live on the same dimension of between-subject variability and are, therefore, affected by measurement reliability in the same way.

The aim of this brief report is therefore (1) to clarify and highlight the relevance of the reliability paradox for studying group differences (2) to present simulation-based illustrations to make the implications of the reliability paradox more intuitive, and (3) to provide a set of mathematical formulae for effect size attenuation that cover different scenarios of group comparisons.

## 2 Methods

### 2.1 Simulated data

To simulate data, we sampled from a normal distribution


(1) $$\begin{array}{l} X\sim N\left(\mu ,\sigma_{b}^{2}+\sigma_{e}^{2}\right) \end{array}$$

by independently varying between-subject ($\sigma_{b}^{2}$) and error ($\sigma_{e}^{2}$) variances. To represent repeated measurements of task performance, we generated two distributions (“test” and “retest”) with the same mean μ = 0. To simulate one-sample effects, we simply generated another distribution that is shifted upward by a constant offset (μ = 2). To simulate paired-sample effects, we generated two distributions (corresponding to Condition 1 and Condition 2) one of which was at μ = 0 and the other at μ = 2. Finally, to illustrate relationships with external traits, we generated additional datasets with fixed between-subject variance ($\sigma_{b}^{2}$) and no error variance ($\sigma_{e}^{2}=0$). We specified true population correlations of *r*_*true*_ = 0.5 and *r*_*true*_ = 0.9 to represent different levels of association between task performance and symptom/trait measures.

Note, while we refer to these data distributions as representing “task performance” and “traits/symptoms” to make this analysis more intuitive, these datasets are generated at a high level of abstraction and do not assume any specific data-generating process—i.e., we are not simulating trial-level or item-level data, we are simply generating distributions of individual-level scores.

Patients vs. controls groups were created by splitting the datasets such that 10% of the distribution with the highest symptom scores were assigned to the patient group while the remaining 90% were assigned to the control group. For creating high vs. low trait groups, we simply performed a median split across the datasets.

To achieve sufficient stability of the test-retest reliability and effect size estimates, we used a sample size of 10,000 for each combination of σ_*b*_ and σ_*e*_, each of which was varied between 0.3 and 2 for most of the analysis. To further increase the stability of our effects, when investigating the relationship between true and observed effect size metrics as a function of reliability, we increased the sample size to 1,000,000. We also kept between-subject variance fixed at σ_*b*_ = 0.5, and only varied error variance in the range σ_*e*_∈[0.01 3]. When comparing how the different statistical metrics fare when it comes to significance testing, we used a sample of *N* = 60 (a typical effect size seen in practice)—to achieve stable estimates of *p*-values we averaged results over 20,000 repetitions.

### 2.2 Statistical analysis

To assess test-retest reliability, we used the intraclass correlation coefficient (ICC) (Fleiss, 2011; Koo and Li, 2016; Liljequist et al., 2019):


(2) $$\begin{array}{l} ICC=\frac{\sigma_{b}^{2}}{\sigma_{b}^{2}+\sigma_{e}^{2}}, \end{array}$$

where for clarity we omit the within-subject variance term in the denominator because throughout our analysis it was kept at 0 between test and retest measurements.

To measure group effects, we used Cohen's *d* as the main effect size metric for both within- and between-group effects. To account for unequal variances between groups when performing 90/10% split (for controls vs. patients), we used d*, a variant of Cohen's *d* that does not assume equal variances:


(3) $$\begin{array}{l} d=\frac{\mu_{2}-\mu_{1}}{\sigma_{p}},\;\text{where}\\ \sigma_{p}=\sqrt{\frac{\left(n_{1}-1\right)\sigma_{1}^{2}+\left(n_{2}-1\right)\sigma_{2}^{2}}{n_{1}+n_{2}-2}} \end{array}$$

(4) $$\begin{array}{l} d^{*}=\frac{\mu_{2}-\mu_{1}}{\sigma_{np}},\;\text{where }\sigma_{np}=\sqrt{\frac{\sigma_{1}^{2}+\sigma_{2}^{2}}{2}} \end{array}$$

Equation 3 is the standard method for calculating Cohen's *d* using the pooled standard deviation, where in the numerator we have the difference between the means of the two groups (μ_1_ and μ_2_), with *n*_1_ and *n*_2_ denoting the sample sizes of each group, and $\sigma_{1}^{2}$ and σ^2^ denoting the variances of each group. In contrast, Cohen's d* (Equation 4) is based on the non-pooled standard deviation. While the use of a non-pooled standard deviation somewhat complicates the interpretation of the resulting effect size metric, empirical investigations have shown that it possesses robust inferential properties and may be a more practical option given that the equal variance requirement is rarely met in practice (Delacre et al., 2021).

To be more comprehensive in our analysis, alongside Cohen's *d*, we also report a non-parametric alternative, the rank-biserial correlation coefficient (*r*_*rb*_). To perform the associated statistical significance tests, we use the *t*-test and the Mann-Whitney U test, respectively.

To quantify the impact of reliability on statistical power, we calculated the required sample sizes for each effect size metric to achieve 80% power at α = 0.05 (Cohen, 2013). The critical values *z*_α/2_ and *z*_β_ are defined as:


(5) $$\begin{array}{l} z_{\alpha /2}=\Phi^{-1}\left(1-\alpha /2\right)\;\text{and }z_{\beta }=\Phi^{-1}\left(1-\beta \right), \end{array}$$

where Φ^−1^ is the inverse cumulative distribution function of the standard normal distribution, α = 0.05 is the two-sided significance level, and β = 0.20 (corresponding to 80% power) is the Type II error rate. With these parameters, *z*_α/2_ = 1.96 and *z*_β_ = 0.84.

For Pearson correlation, we used the Fisher z-transform approach (Cohen, 2013):


(6) $$\begin{array}{l} N_{r}=3+{\left(\frac{z_{\alpha /2}+z_{\beta }}{\text{atanh}\left(\left|r_{obs}\right|\right)}\right)}^{2}, \end{array}$$

where |*r*_*obs*_| is the absolute value of the observed correlation attenuated by measurement error. For Cohen's *d* from median split analysis, we used the standard two-sample *t*-test power formula (Cohen, 2013):


(7) $$\begin{array}{l} N_{d}=4{\left(\frac{z_{\alpha /2}+z_{\beta }}{d_{obs}}\right)}^{2}, \end{array}$$

where *d*_*obs*_ is the observed effect size attenuated by reliability. For the rank-biserial correlation with equal group sizes, we used (Noether, 1987):


(8) $$\begin{array}{l} N_{rb}\approx \frac{4}{3}{\left(\frac{z_{\alpha /2}+z_{\beta }}{r_{rb,obs}}\right)}^{2}, \end{array}$$

where *r*_*rb, obs*_ is the observed rank-biserial correlation coefficient.

## 3 Results

### 3.1 The reliability paradox

**Figure 1:**
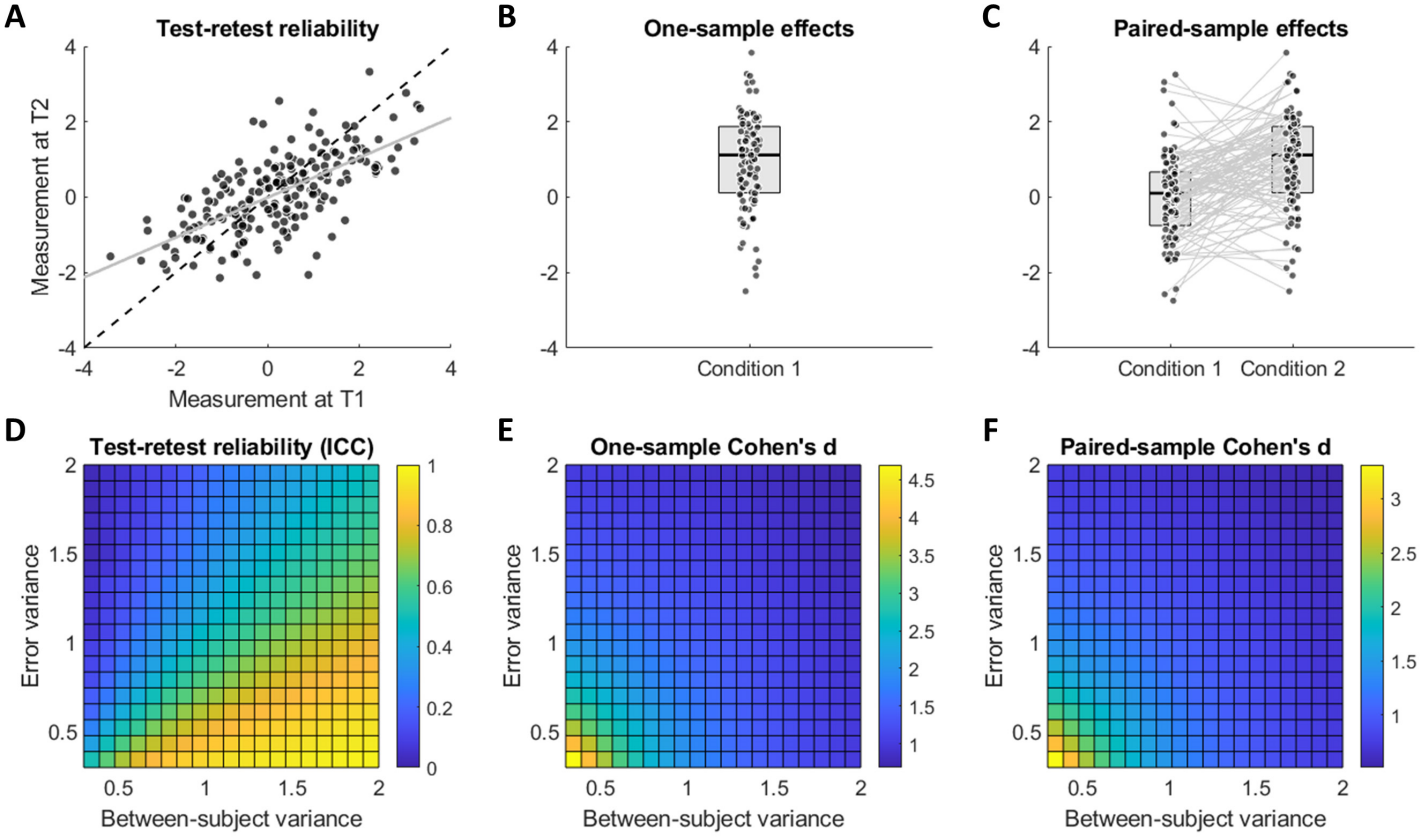
The reliability paradox. Top panels **(A–C)** illustrate the statistical tests under consideration: **(A)** test-retest correlation (same measure obtained twice), **(B)** one-sample test (mean of a single condition compared to zero), and **(C)** paired-sample test (mean difference between two conditions). Bottom panels **(D–F)** show how the observed outcomes of these tests depend on the relative contributions of error variance and between-subject variance. Test-retest reliability **(D)** increases when error variance is minimized and between-subject variance is maximized, whereas observed one-sample and paired-sample effect sizes **(E, F)** increase when both error and between-subject variances are minimized.

First, we performed data simulations to illustrate the reliability paradox—namely, that strong within-group effects are inherently at odds with high test-retest reliability. We generated multiple sets of synthetic data by independently varying between-subject variance ($\sigma_{b}^{2}$) and measurement error variance ($\sigma_{e}^{2}$), and explored how this affects test-retest reliability and observed within-group effects (Figure 1). The key takeaway here is that while test-retest reliability is determined by the proportion of between-subject variance relative to total variance $\sigma_{b}^{2}/\left(\sigma_{b}^{2}+\sigma_{e}^{2}\right)$, within-group effects depend on the total variance $\sigma_{b}^{2}+\sigma_{e}^{2}$. In other words, increasing error variance $\sigma_{e}^{2}$ will reduce both reliability and within-group effect sizes, whereas increasing between-subject variance $\sigma_{b}^{2}$ will improve reliability but will reduce within-group effects, since a fixed mean difference becomes smaller relative to the larger total variance.

Note that for simplicity here we assumed the between-subject variance in condition 1 and condition 2 to be uncorrelated (Figure 1C). Under this assumption, the variance of the difference scores (i.e., the individual-level condition differences used to compute paired-sample Cohen's d) is equal to the sum of the variances in each condition. Due to this linear relationship, Figure 1F can therefore be interpreted as referring to the between-subject variance of difference scores. In practice, however, task conditions are often positively correlated, which reduces the variance of the difference scores—thereby inflating effect sizes while reducing the reliability of the underlying scores (Cronbach and Furby, 1970; Hedge et al., 2018; Draheim et al., 2019). This would introduce non-linearities in how the between-subject variance of each condition relates to the observed effect size, but the relationship between the between-subject variance of difference scores and the observed effect size, which we aim to convey here, still holds.

### 3.2 Group differences: data simulations

#### 3.2.1 Data simulations for groups created by dichotomizing continuous measures

**Figure 2:**
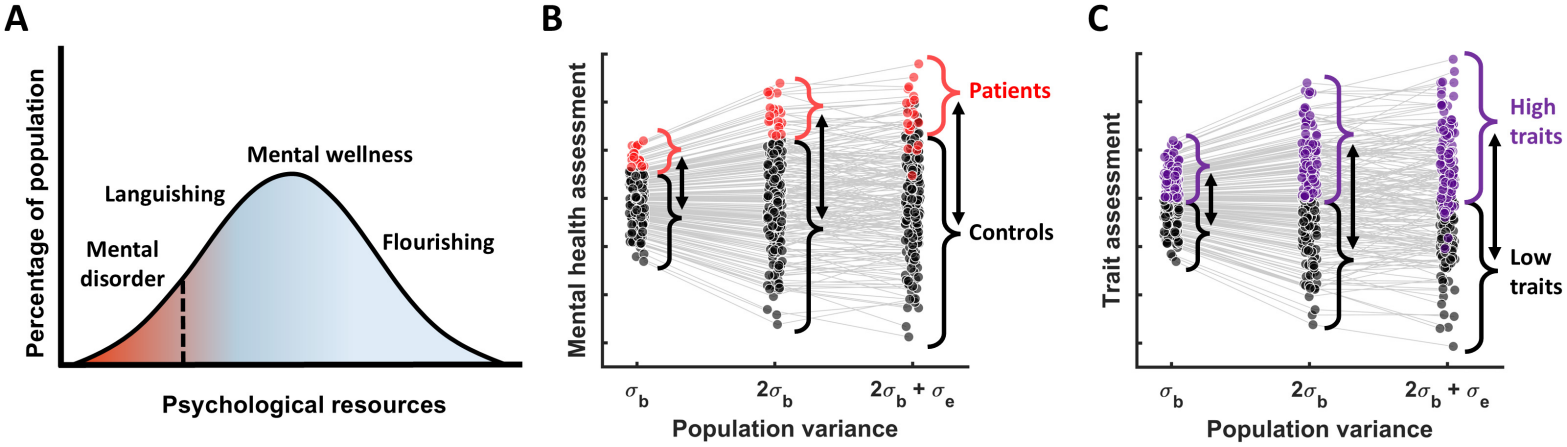
Group differences as a function of population variance. **(A)** The dimensional view of mental disorders; adapted from Huppert et al. (2005). **(B)** The relationship between patients vs. controls group differences and population variance, assuming that patients are defined as 10% of the population with the poorest mental health. **(C)** A more general case illustrating the group differences resulting from the median split of the data (based on some cognitive measure) as a function of population variance. In both **(B)** and **(C)** we see that as true population variance increases, raw group difference increase too, while adding measurement error to the true scores results in misclassification of some individuals—which will end up attenuating observed group differences in the measures of interest.

Next, using data simulations we investigated how measurement reliability affects group differences when the groups are derived by thresholding a continuous measure (e.g., symptoms or traits). To make this more intuitive we considered two common scenarios: 1) mental disorders, which can be generally thought of as occupying the low end of the wellbeing distribution (Huppert et al., 2005) or any specific symptom dimension, and 2) “low” vs. “high” cognitive traits formed by a median split (Figure 2). Considering these scenarios using simulations helps illustrate one key insight: raw group differences scale together with between-subject variance (Figures 2B, C). Hence, unlike with within-group effects, reducing between-subject variance does not lead to larger group effects. Adding measurement error can further increase raw group differences, but it also leads to misclassification (Figures 2B, C), which ultimately reduces observed group differences in any other measures of interest, as we will see next.

**Figure 3:**
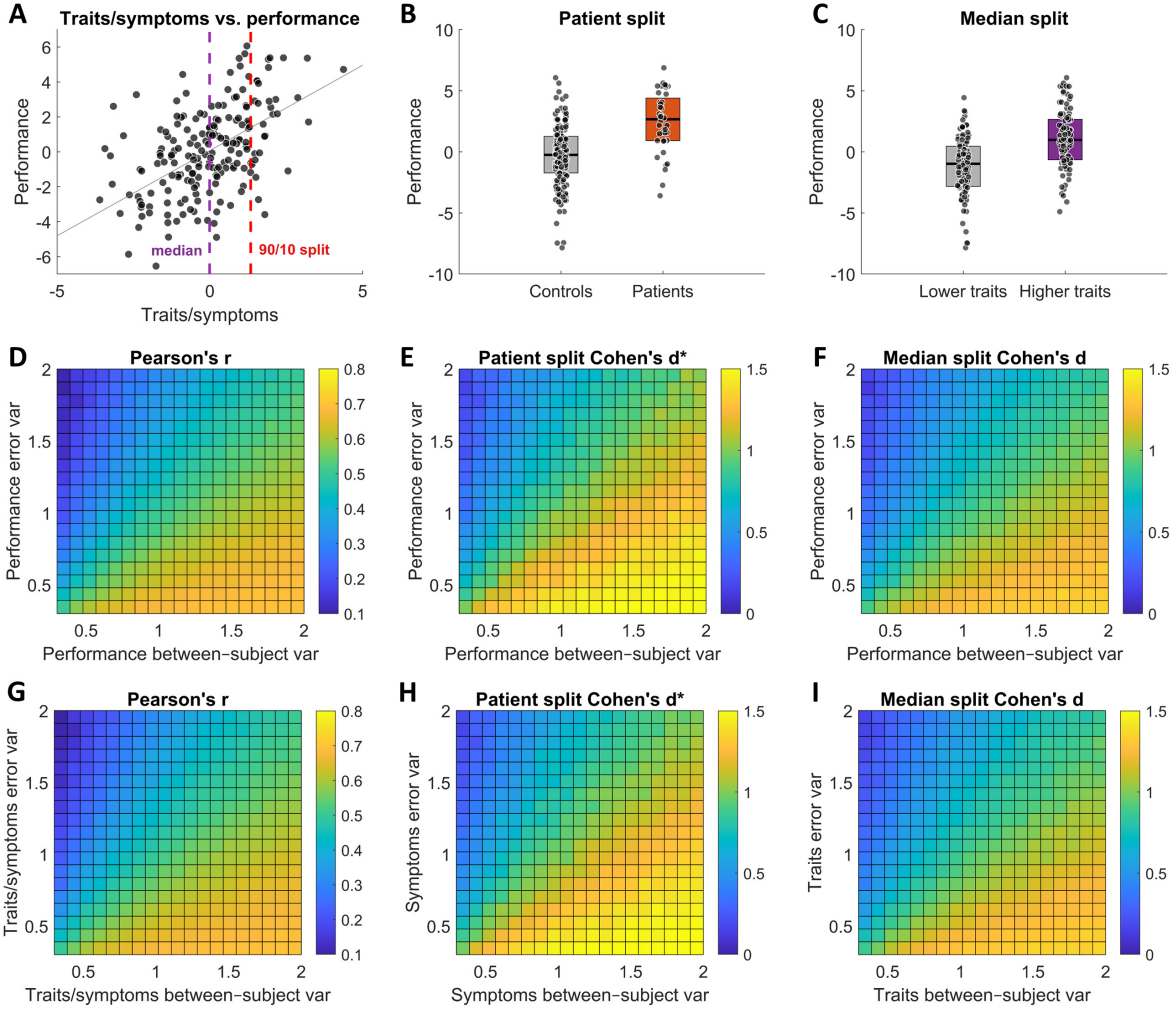
Test-retest reliability effects on observed group differences. The top row panels **(A–C)** illustrate the different analysis scenarios, while the 2 row panels **(D–F)** show the corresponding observed effects for different error and between-subject variance values of task performance, and the bottom row panels **(G–I)** show the corresponding observed effects for different error and between-subject variance values of symptoms/traits. **(A)** An illustration of correlation between traits or symptoms and task performance. The vertical dashed lines indicate how the data was split for the two analysis scenarios. **(B)** An illustration of patient and control groups created by assigning 10% of the population with the poorest mental health to patient group and the remaining 90% to control group. **(C)** An illustration of “low” and “high” trait groups by performing a median split. Overall, the test results show that both observed correlation strength and observed group differences increase with increasing test-retest reliability (i.e., with reducing error variance/increasing between-subject variance) both when varying between-subject and error variances of task performance measures **(D–F)** and symptoms/traits measures **(G–I)**.

We generated correlated “symptoms/traits” and “task performance” datasets such that they had *r*_*true*_ = 0.7 Pearson's correlation. To derive the groups, we defined patients as occupying the 10% of the population with the poorest mental health (Figures 3A, B), with the rest of the population being controls; using the median split along the trait dimension Figure 3A, we grouped individuals into “low” and “high” trait groups (Figure 3C).

We then examined how the observed effect size metrics were affected by independently varying σ_*b*_ and σ_*e*_ of task performance and then of traits/symptoms (**see Methods for more details**). In both cases, we find the same results: reducing reliability in either task performance measures (Figures 3D–F) or symptoms/traits measures (Figures 3G–I) leads to attenuation of observed effect sizes that mirror those of correlational analyses of individual differences (Equation 9).

#### 3.2.2 Comparing attenuation effects across effect size metrics

In a correlational analysis, the true correlation strength between a measure *x* and a measure *y* is attenuated by their respective reliabilities following (Spearman, 1904):


(9) $$\begin{array}{l} r_{observed}=r_{true}\sqrt{ICC_{x}ICC_{y}}. \end{array}$$

**Figure 4:**
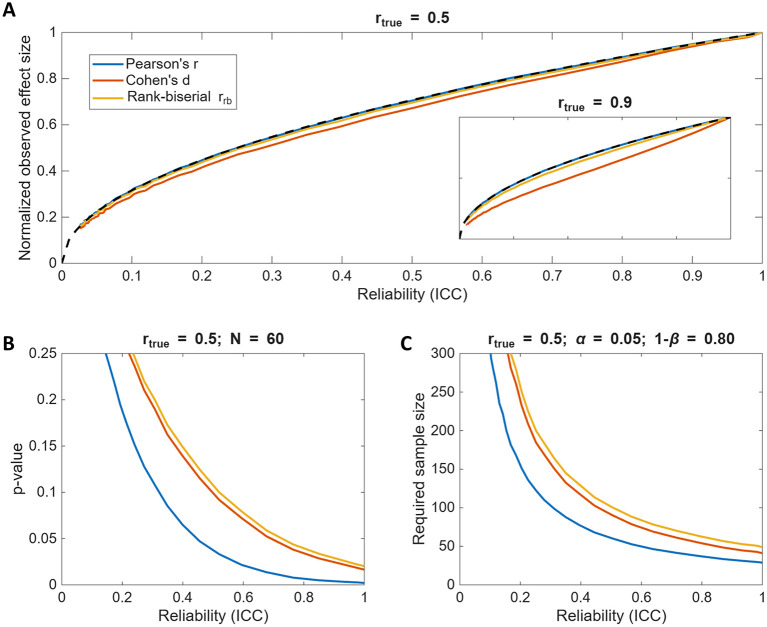
Test-retest reliability effects across different effect size metrics and statistical tests. **(A)** The observed effect sizes as a function of reliability for *r*_*true*_ = 0.5, comparing group differences to correlational strength. Note, because the effect sizes among the tests are not directly comparable, each effect size is normalized by its own maximum value at ICC = 1. The inset shows the results for *r*_*true*_ = 0.9. The dashed line denotes $r_{observed}=r_{true}\sqrt{ICC_{x}ICC_{y}}$. **(B)** The *p*-value as a function of reliability for *r*_*true*_ = 0.5 and the total sample size of *N* = 60. Dichotomizing data substantially increases *p*-values, especially when reliability is low. **(C)** The required sample size to achieve 80% statistical power at α = 0.05 as a function of reliability for the three effect size metrics. Dichotomizing data substantially increases the required sample sizes to detect the same true effect, especially when reliability is low.

We compared the observed effect sizes from our simulations to the predicted attenuation relationship and found that both parametric (Cohen's *d*) and non-parametric (rank-biserial *r*_*rb*_) estimates closely followed the same reliability-based scaling as Pearson's *r*, especially for moderate true correlations (*r*_true_ = 0.5; Figure 4A). However, Cohen's *d* deviated more substantially when *r*_true_ = 0.9 (Figure 4A) inset due to increasing deviations from normality caused by dichotomization. Thus, when the assumptions of the effect size metric hold, observed between-group differences can be approximated as:


(10) $$\begin{array}{l} \delta_{\text{observed}}=\delta_{\text{true}}\sqrt{ICC_{x}ICC_{y}} \end{array}$$

Although attenuation similarly affects both correlations and group differences, it is important to keep in mind that correlational analyses generally retain greater statistical power. Figure 4B illustrates that *p*-values for group comparisons of dichotomized data are larger than those for correlation tests (*N* = 60, *r*_true_ = 0.5) and Figure 4C similarly illustrates that required sample sizes (to have 80% power at α = 0.05) for dichotomized data are substantially larger than those for correlational analysis, especially when reliability is low. That is simply because the variance discarded during dichotomization results in information loss. This is well documented in previous work (e.g., MacCallum et al., 2002; Royston et al., 2006; Naggara et al., 2011; Streiner, 2002) therefore we will not go into any further details here. Just, please, avoid dichotomizing your continuous data as much as you can.

### 3.3 Group differences: mathematical derivations

#### 3.3.1 Attenuation for exogenously defined groups

In previous sections, we used simulations to illustrate how poor reliability attenuates group differences when groups are derived from a noisy continuous measure. These visualizations were meant to provide an intuitive understanding of the attenuation effect. Here, we derive the same effect mathematically, but this time considering exogenously defined groups (e.g., male vs. female or treatment vs. control), which are categorical and not subject to measurement error.

For such externally defined groups, random measurement error does not systematically bias the raw mean difference (Lord and Novick, 1968; Nunnally and Bernstein, 1994) but inflates total variance ($\sigma_{b}^{2}+\sigma_{e}^{2}$). Consequently, the raw difference scales with between-subject variance rather than total variance (Cohen, 1988; Ellis, 2010):


(11) $$\begin{array}{l} \mu_{2}-\mu_{1}=\delta_{\text{true}}\;\sigma_{b}. \end{array}$$

The expression for δ_*observed*_ will then depend on total variance, such that:


(12) $$\begin{array}{l} \delta_{\text{observed}}=\frac{\mu_{2}-\mu_{1}}{\sqrt{\sigma_{b}^{2}+\sigma_{e}^{2}}} \end{array}$$

(13) $$\begin{array}{l} =\delta_{\text{true}}\frac{\sigma_{b}}{\sqrt{\sigma_{b}^{2}+\sigma_{e}^{2}}} \end{array}$$

(14) $$\begin{array}{l} =\delta_{\text{true}}\sqrt{ICC}\;, \end{array}$$

where used Equations 11, 2 to get to the final expression.

Notably, this same attenuation mechanism applies to both externally defined groups and groups formed via dichotomization, although the latter additionally suffers from misclassification effects.

#### 3.3.2 Attenuation when the groups have different reliabilities

Equation 14 assumes both groups have the same measurement reliability, but this is not always the case. Here, we derive a more general formula that accounts for differing reliabilities. If the groups have reliabilities *ICC*_1_ and *ICC*_2_, their observed standard deviations are:


(15) $$\begin{array}{l} \sigma_{1}=\frac{\sigma_{b}}{\sqrt{ICC_{1}}},\;\sigma_{2}=\frac{\sigma_{b}}{\sqrt{ICC_{2}}}, \end{array}$$

where we assume both groups share the same true between-subject standard deviation. Using the non-pooled variance estimation (see Methods for more details), the observed standard deviation is given by:


(16) $$\begin{array}{l} \sigma_{np}=\sqrt{\frac{\sigma_{1}^{2}+\sigma_{2}^{2}}{2}}=\sqrt{\frac{\sigma_{b}^{2}}{2}\left(\frac{1}{ICC_{1}}+\frac{1}{ICC_{2}}\right)}\\ \;=\sigma_{b}\sqrt{\frac{1}{2}\left(\frac{1}{ICC_{1}}+\frac{1}{ICC_{2}}\right)}. \end{array}$$

Thus, the observed standardized difference is:


(17) $$\begin{array}{l} \delta_{\text{observed}}=\frac{\mu_{2}-\mu_{1}}{\sigma_{np}} \end{array}$$

(18) $$\begin{array}{l} =\frac{\delta_{\text{true}}\sigma_{b}}{\sigma_{b}\sqrt{\frac{1}{2}\left(\frac{1}{ICC_{1}}+\frac{1}{ICC_{2}}\right)}} \end{array}$$

(19) $$\begin{array}{l} =\delta_{\text{true}}\sqrt{\frac{2}{\frac{1}{ICC_{1}}+\frac{1}{ICC_{2}}}} \end{array}$$

(20) $$\begin{array}{l} =\delta_{\text{true}}\sqrt{\frac{2ICC_{1}ICC_{2}}{ICC_{1}+ICC_{2}}}. \end{array}$$

In the special case where *ICC*_1_ = *ICC*_2_ = *ICC*, this expression simplifies to Equation 14, consistent with the earlier result.

#### 3.3.3 Attenuation when true variances are also unequal

Thus far, we assumed that both groups share the same underlying between-subject standard deviation. Here, we relax that assumption and allow the two groups to have different true variances, such that the total true variance is


(21) $$\begin{array}{l} \sigma_{b,\text{np}}=\sqrt{\frac{\sigma_{b,1}^{2}+\sigma_{b,2}^{2}}{2}}\;, \end{array}$$

and the observed variances are


(22) $$\begin{array}{l} \sigma_{\text{obs},1}=\frac{\sigma_{b,1}}{\sqrt{ICC_{1}}},\;\sigma_{\text{obs},2}=\frac{\sigma_{b,2}}{\sqrt{ICC_{2}}}\;, \end{array}$$

and so the total observed unpooled variance is


(23) $$\begin{array}{l} \sigma_{\text{np}}=\sqrt{\frac{\sigma_{\text{obs},1}^{2}+\sigma_{\text{obs},2}^{2}}{2}}=\sqrt{\frac{\sigma_{b,1}^{2}/ICC_{1}+\sigma_{b,2}^{2}/ICC_{2}}{2}}. \end{array}$$

Thus, the observed standardized difference is


(24) $$\begin{array}{l} \delta_{\text{observed}}=\frac{\mu_{2}-\mu_{1}}{\sigma_{\text{np}}}=\frac{\delta_{\text{true}}\sigma_{b,\text{np}}}{\sigma_{\text{np}}}. \end{array}$$

Substituting Equations 21, 23 into Equation 24 yields


(25) $$\begin{array}{l} \delta_{\text{observed}}=\delta_{\text{true}}\sqrt{\frac{\sigma_{b,1}^{2}+\sigma_{b,2}^{2}}{\sigma_{b,1}^{2}/ICC_{1}+\sigma_{b,2}^{2}/ICC_{2}}}. \end{array}$$

In the special case where σ_*b*, 1_ = σ_*b*, 2_ = σ_*b*_, Equation 25 simplifies to Equation 20.

#### 3.3.4 Attenuation by classification reliability

We can further extend these equations to take into account the reliability of group labels (e.g., patients vs. controls). Note, that we have already demonstrated via simulations that when group classification is error prone, the observed group differences scales as $\sqrt{ICC}$ for the underlying continuous measure. However, when comparing two groups, a more likely measure of classification reliability that would be used is Cohen's Kappa (κ) (Cohen, 1960), which measures the reliability of categorical labels (and is often used to quantify the inter-rater reliability of clinical diagnoses). The relationship between κ and the underlying reliability of continuous measures *ICC* can be shown to be Kraemer (1979):


(26) $$\begin{array}{l} \kappa =\frac{2}{\pi }\arcsin\left(\sqrt{ICC}\right). \end{array}$$

Rearranging this for *ICC* gives


(27) $$\begin{array}{l} ICC=\sin^{2}\left(\frac{\pi }{2}\kappa \right). \end{array}$$

Now, the expression Equation 10 can be re-expressed in terms of classification reliability, while Equations 20, 25 can be further extended to account for classification reliability:


(28) $$\begin{array}{l} \delta_{\text{observed}}=\delta_{\text{true}}\sqrt{ICC\cdot \sin\left(\frac{\pi }{2}\kappa \right)}, \end{array}$$

(29) $$\begin{array}{l} \delta_{\text{observed}}=\delta_{\text{true}}\sqrt{\frac{2ICC_{1}ICC_{2}}{ICC_{1}+ICC_{2}}\sin\left(\frac{\pi }{2}\kappa \right)}, \end{array}$$

(30) $$\begin{array}{l} \delta_{\text{observed}}=\delta_{\text{true}}\sqrt{\frac{\sigma_{b,1}^{2}+\sigma_{b,2}^{2}}{\sigma_{b,1}^{2}/ICC_{1}+\sigma_{b,2}^{2}/ICC_{2}}\sin\left(\frac{\pi }{2}\kappa \right)}. \end{array}$$

We summarize all the attenuation equations in Box 1.

**Box 1:** Attenuation of observed group differences in different scenarios.

$\delta_{\text{observed}}=\delta_{\text{true}}\sqrt{ICC}$	Continuous outcome measured with reliability *ICC*; classification is error-free.
$\delta_{\text{observed}}=\delta_{\text{true}}\sqrt{ICC\cdot \sin\left(\frac{\pi }{2}\kappa \right)}$	Continuous outcome measured with reliability *ICC* while classification reliability is κ.
$\delta_{\text{observed}}=\delta_{\text{true}}\sqrt{\frac{2ICC_{1}ICC_{2}}{ICC_{1}+ICC_{2}}}$	Continuous outcome with group-specific reliabilities *ICC*_1_ and *ICC*_2_; classification is error-free.
$\delta_{\text{observed}}=\delta_{\text{true}}\sqrt{\frac{2ICC_{1}ICC_{2}}{ICC_{1}+ICC_{2}}\sin\left(\frac{\pi }{2}\kappa \right)}$	Continuous outcome measured with group-specific reliabilities *ICC*_1_ and *ICC*_2_ while classificaiton reliability is κ.
$\delta_{\text{observed}}=\delta_{\text{true}}\sqrt{\frac{\sigma_{b,1}^{2}+\sigma_{b,2}^{2}}{\sigma_{b,1}^{2}/ICC_{1}+\sigma_{b,2}^{2}/ICC_{2}}}$	Continuous outcome with group-specific variances $\sigma_{b,1}^{2}$, $\sigma_{b,2}^{2}$ and reliabilities *ICC*_1_, *ICC*_2_; classification is error-free.
$\delta_{\text{observed}}=\delta_{\text{true}}\sqrt{\frac{\sigma_{b,1}^{2}+\sigma_{b,2}^{2}}{\sigma_{b,1}^{2}/ICC_{1}+\sigma_{b,2}^{2}/ICC_{2}}\sin\left(\frac{\pi }{2}\kappa \right)}$	Continuous outcome measures with with group-specific variances $\sigma_{b,1}^{2}$, $\sigma_{b,2}^{2}$ and reliabilities *ICC*_1_, *ICC*_2_, while classification reliability is κ.

## 4 Discussion

This report extends the implications of the reliability paradox beyond its original focus on individual differences (Hedge et al., 2018), demonstrating that it presents the same problems when studying group differences. When groups are formed by thresholding continuous measures (e.g., patients vs. controls), the resulting loss of statistical power makes detecting group differences (vs. individual differences) even harder when reliability is low. We hope that this work will help raise awareness of measurement reliability implications in group differences research and that the provided mathematical expressions will help researchers better account for the magnitude of the effect size attenuation in their studies.

### 4.1 Implications for clinical translation

Poor reliability leads to small observed effects, which severely impedes clinical translation (Karvelis et al., 2023; Nikolaidis et al., 2022; Gell et al., 2023; Tiego et al., 2023; Moriarity and Alloy, 2021; Paulus and Thompson, 2019; Hajcak et al., 2017). For example, for a measure to have diagnostic utility—defined as ≥80% sensitivity and ≥80% specificity—it must show a group difference of *d*≥1.66 (Loth et al., 2021). Note that *d*≥0.8 is considered “large” and it is rarely seen in practice. This may also explain why treatment response prediction research, where it is common to dichotomize symptom change into responders vs. non-responders, has so far shown limited success (Karvelis et al., 2022). Improving the reliability of measures to uncover the landscape of large effects is therefore of paramount importance (DeYoung et al., 2025; Nikolaidis et al., 2022; Zorowitz and Niv, 2023). This applies not only to cognitive performance measures—where the reliability paradox discussion originates—but equally to other instruments including clinical rating scales and diagnostic criteria (Regier et al., 2013; Shrout, 1998), self-report questionnaires (Enkavi et al., 2019; Vrizzi et al., 2025), and experience sampling methods (ESM) (Dejonckheere et al., 2022; Csikszentmihalyi and Larson, 1987). To begin uncovering large effect sizes, however, reliability analysis and reporting must first become a routine research practice (Karvelis et al., 2023; Parsons et al., 2019; LeBel and Paunonen, 2011). While some guidelines such as APA's JARS for psychological research (Appelbaum et al., 2018) and COSMIN for health measurement instruments (Mokkink et al., 2010) do encourage routine reporting of reliability, others, such as PECANS for cognitive and neuropsychological studies (Costa et al., 2025), do not mention reliability or psychometric quality at all, underscoring the need to continue raising awareness of measurement reliability issues.

### 4.2 Double bias: reliability attenuation and small-sample inflation

Correct interpretation of observed effects requires considering not only the attenuation effects we describe here, but also sampling error. While low measurement reliability attenuates observed effect sizes, small samples produce unstable estimates that are often selectively reported, leading to systematic inflation of reported effects—known as the winner's curse (Sidebotham and Barlow, 2024; Button et al., 2013; Ioannidis, 2008). Currently, research in cognitive neuroscience and psychology is dominated by small samples, with an estimated 50% of research reporting false positive results (Szucs and Ioannidis, 2017); also see Schäfer and Schwarz (2019). While the attenuation of effect sizes can be addressed by the equations we provide, inflation due to the winner's curse can be mitigated by collecting larger samples, preregistering analyses, applying bias-aware estimation or meta-analytic techniques (Button et al., 2013; Nosek et al., 2018; Zöllner and Pritchard, 2007; Vevea and Hedges, 1995).

### 4.3 Broader implications for real-world impact

Although we presented our statistical investigation with psychiatry and cognitive sciences in mind, the implications of our results are quite general and could inform any area of research that relies on group comparisons, including education, sex, gender, age, race, and ethnicity (e.g., Hyde, 2016; Ones and Anderson, 2002; Roth et al., 2001; Rea-Sandin et al., 2021; Perna, 2005; Vedel, 2016). The reliability of measures is rarely considered in such studies, but the observed effect sizes are often treated as proxies for practical importance (Cook et al., 2018; Funder and Ozer, 2019; Kirk, 2001, 1996; Olejnik and Algina, 2000) and are used to inform clinical practice (e.g., Ferguson, 2009; McGough and Faraone, 2009) and policy (e.g., Lipsey et al., 2012; Pianta et al., 2009; McCartney and Rosenthal, 2000). Not accounting for the reliability of measures can therefore create a very misleading scientific picture and lead to damaging real-world consequences.

### 4.4 Limitations and caveats

Our derivations of effect size attenuation are based on parametric assumptions and may not give precise estimates when the data is highly non-normal or is contaminated with outliers. By extension, they may not give precise estimates for non-parametric group differences metrics, although it should still provide a good approximation. Furthermore, we should highlight once again that our derivations rely on using non-pooled variance for calculating standardized mean differences, which allows dropping the assumption of equal variance. Thus, when the true variances are indeed not equal between the groups, it is important to use the non-pooled variance version of Cohen's d* (see Delacre et al., 2021, for further details) when using the attenuation equations. However, if the true variances are roughly equal, the attenuation relationships derived here will hold just as well for the standard Cohen's d, which uses pooled variance.

## Data Availability

The datasets presented in this study can be found in online repositories. The code for producing the data simulations and figures is available at: https://github.com/povilaskarvelis/clarifying_the_reliability_paradox.
